# Cataphoresis for Obtunding Sensitive Dentin

**Published:** 1896-07

**Authors:** H. L. Ambler

**Affiliations:** Cleveland.


ARTICLE IV.

### CATAPHORESIS FOR OBTUNDING SENSITIVE DENTIN.

DR. H. L. AMBLER, CLEVELAND.

Cataphoresis means "the movement of fluids and the substances they hold in solution, from the positive pole of electrodes conveying a continuous current in tissue (dentin included) toward the negative pole." This method can be applied in nearly all cases, and where the sensitiveness is not overcome by cocain, we use oil of cassia in which has been thoroughly incorporated a trace of thoroughly pulverized soda sulfate. In unusually dense dentin the 25 per cent solution of cocain hydrochlorate penetrates slowly but the action and penetration may be increased by adding a trace of soda sulfate, thus making the conducting power of the liquid greater. During action on the dentine, the current carries with it almost any fluid exposed to its action under proper conditions, that is, any local anesthetic which is a conductor of electricity, or can be made one, is driven or forced into the dentine, depending on the character of the obtundent, porosity of dentin, voltage and length of time it is applied, a high voltage requiring less time, with the same obtundent, than when a low voltage is used. Sometimes the voltage can be carried to twenty or thirty in five minutes without discomfort to the patient. At other times it can be carried to 40 volts in ten minutes. the latter is as high a pressure as we have ever found necessary to use in obtunding sensitive dentin, but when we attempt to bleach devitalized teeth, a much higher voltage becomes necessary. We have only spoked of volts as shown on the index of the "selecter," invented by Dr. Gillette and Mr. George M. Wheeler, as they constitute our guide, when no milliampere meter is used. Extreme vascularity, as found in young patients, and those of lymphatic temperament, will be found to yield more readily to treatment than dense dentin in old teeth, or those of bilious temperament. Large tubuli are more easily and quickly dehydrated than small; large tubuli admit the driving down into them of drugs by electrical action much easier than small ones. Dryness of a cavity is essential, and when the rubber has been applied to the tooth, and the cavity wiped with absorbent cotton, then it is fairly dry, and it is not necessary to attempt further dessication before using the cataphoretic treatment; neither should we do so in extremely sensitive teeth, nor for very nervous patients; because when we apply alcohol as a dehydrant, the sudden cold produced by its rapid evaporation causes pain which might have been avoided, even if it does only last for a minute, still we have been led to think that many times where alcohol was used before applying cataphoresis, the cataphoretic action was more rapid, as the medicament could be forced into the tubuli quicker and easier than when dehydration was not practiced. We are well aware of the value of dehydration, produced hy absolute alcohol and a current of warm air, as an obtundent, but we did not use this process before applying cataphoresis; we simply wiped the cavity with a pellet of cotton saturated with alcohol; though used in this manner, it does not produce much obtundent effect.

The degree of success in obtunding does not seem to depend on the location or size of the cavity, as with some other methods; in a small cavity there can be no danger of injuring the pulp, and in a large cavity, where the pulp is nearly exposed, the medicament acts as an anesthetic, and after ceasing to apply it, in a short time the pulp returns to its normal sensitiveness, and it is the same with sensitive dentin which has been obtunded; of course, the time required largely depends on vitality, physical characteristics, length of time applied, kind of obtundent used, etc. Results vary, and when cutting rapidly you are liable at any time to pass through the obtunded portion, but should this happen, a second application can be made. Experiments have shown that the alternating current will accomplish cataphoresis, but it does not work as quickly or penetrate as deeply as the continuous current, because of the interrupted or wave-like current in alternation; the continuous (direct) current is the most efficient and economical, but the expense connected with either is small. The electric current alone does not produce anesthesia at either terminal but when a proper medicament is placed in the cavity, the current diffuses it through the tooth tissue by the anode (positive pole), but not by the cathode (negative pole). The negative pole is so related to the positive pole that the current passing through the tooth, from the positive pole to the negative pole, and thus obtunding, is accomplished at the point to be operated on. The excavating should be commenced as soon as the positive electrode (platinum) has been removed from the cavity, and if you cut through the desensitized layer before the cavity is properly formed, and the patient will not allow you to proceed, then another application can be made and another layer of dentin desensitized, thus enabling you to complete the excavating without pain. By applying for a sufficient length of time we think any cavity can be obtunded.

We are using the Fractional Volt Selecter (manufactured by the Electro-Therapeutic Company; 32 East Twenty-third street, New York) attached to the one hundred and ten volt continuous current. The selecter is adapted, when made, to either the one hundred and ten volt continuous or alternating current; also the arc lighting circuit can be used by putting in a transformer to reduce the voltage before it reaches the selecter, which is reliable and delicate as a mechanical apparatus; we have tested the voltage with a volt-meter, and find it to be satisfactory. The volt-meter is used to determine the difference of potential between any two points of a circuit by connecting its terminals as a shunt to the circuit between these two points. The selecter regulates the voltage of electric current, and accomplishes with exactness a positive control of these currents in very small gradations at the will of the operator, with no sudden increase of voltage, but a continuous, smooth, increase of voltage, imperceptible to sensitive nerves; it also controls the current from a battery in the same manner as when using the incandescent or arc lighting circuit.

Most batteries are not of sufficient strength for cataphoric work on dentin, but a special one for dentists, physicians and surgeons is manufactured, which gives a continuous current from one up to eighty volts. To increase the strength of ordinary batteries, a large number of cells would be necessary, thus making them expensive. A switch has been added to the selecter, which enables the operator to increase the volts from forty to eighty. As first manufactured, only forty volts could be given, but it was found necessary to have more where patients present an unusual resistance to electricity, or when used by physicians and surgeons on parts of the body where the resistance is great. A high pressure of the electric current is necessary to drive the obtundent or medicine into the tissues through thick flesh deeply to the seat of affected parts, and for forcing a bleaching fluid or obtundant through sound dense dentin. Cataphoresis is a recognized scientific process for administering remedies for many diseases, and for surgical operations. * * *

A 25 per cent solution of cocain hydrochlorate, containing 2 per cent boric acid, makes a good conducting media and a stable solution, and good results can be obtained by its use in obtunding sensitive dentin. We have never seen any unpleasant or toxic effects from the use of cocain in the teeth, and it seems safe to use as far as its effect on tooth structure, pulp, or system is concerned. Cocain imprudently administered by other methods to patients of extreme susceptibility, or to hysterical persons, and those in whom the brain or circulatory system is not in a normal condition, may produce general symptoms more or less grave and frequent. In poisoning, from its use, give hypodermic injections of whisky, ether or atropia, and inhalations of amyl nitrit. * * *

Dr. Gillette, to whom great credit is due for having made this process practical, says: "That patients often confuse the sensations of the current with pain."

After the application has been completed, when very sensitive to the current, we turn the index needle on the selecter back to "O," and then remove the sponge electrode first, to prevent shock, but generally these precautions are not necessary, neither are they if you use the latest improved selecter. The running of elevators connected with your current does not produce any appreciable difference, and we have not found it to be detrimental. The electric current is used successfully on the mucous or cutaneous surface for surgical operations when the voltage is controlled by the selecter, and the electric plugger, mouth lamp, cautery, root drier, etc., will probably be adapted to this instrument.—*Ohio Dental Journal.*

